# Nutlin-3a, an MDM2 antagonist and p53 activator, helps to preserve the replicative potential of cancer cells treated with a genotoxic dose of resveratrol

**DOI:** 10.1007/s11033-013-2602-7

**Published:** 2013-05-11

**Authors:** Artur Zajkowicz, Małgorzata Krześniak, Iwona Matuszczyk, Magdalena Głowala-Kosińska, Dorota Butkiewicz, Marek Rusin

**Affiliations:** 1 Center for Translational Research and Molecular Biology of Cancer, Maria Skłodowska-Curie Memorial Cancer Center and Institute of Oncology, Gliwice Branch, ul. Wybrzeże Armii Krajowej 15, 44-101 Gliwice, Poland; 2 Department of Bone Marrow Transplantation, Maria Skłodowska-Curie Memorial Cancer Center and Institute of Oncology, Gliwice Branch, 44-101 Gliwice, Poland

**Keywords:** p53, Nutlin-3a, WIP1, Resveratrol, Senescence

## Abstract

Resveratrol is a natural compound that has been intensely studied due to its role in cancer prevention and potential as an anti-cancer therapy. Its effects include induction of apoptosis and senescence-like growth inhibition. Here, we report that two cancer cell lines (U-2 OS and A549) differ significantly in their molecular responses to resveratrol. Specifically, in U-2 OS cells, the activation of the p53 pathway is attenuated when compared to the activation in A549 cells. This attenuation is accompanied by a point mutation (458: CGA→TGA) in the *PPM1D* gene and overexpression of the encoded protein, which is a negative regulator of p53. Experimentally induced knockdown of *PPM1D* in U-2 OS cells resulted in slightly increased activation of the p53 pathway, most clearly visible as stronger phosphorylation of p53 Ser^37^. When treated with nutlin-3a, a non-genotoxic activator of p53, U-2 OS and A549 cells both responded with substantial activation of the p53 pathway. Nutlin-3a improved the clonogenic survival of both cell lines treated with resveratrol. This improvement was associated with lower activation of DNA-damage signaling (phosphorylation of ATM, CHK2, and histone H2AX) and higher accumulation of cells in the G1 phase of the cell cycle. Thus, the hyperactivation of p53 by nutlin-3a helps to preserve the replicative potential of cells exposed to resveratrol.

## Introduction

In vivo studies have revealed that resveratrol delays cancer development and improves the health of mice on a high-calorie diet [1, 2]. Resveratrol is also considered to be an anti-aging molecule, although different groups have published discordant results on this issue (reviewed in [3]). In vitro experiments with cancer cell lines indicate that resveratrol can induce cell-cycle inhibition, senescence-like growth inhibition, or apoptosis [4]. The mechanistic explanation of results from animal studies are unsatisfactory, partially because of the different concentrations of resveratrol present in the extracellular matrix of target cells of experimental animals and in cancer cell lines treated with resveratrol in vitro (reviewed in [5]).

At concentrations in the range 40–80 μM, resveratrol activates DNA-damage signaling, e.g., phosphorylation of histone H2AX on Ser^139^ [6–8], which can be phosphorylated by DNA damage-activated kinases [9]. A recently published study showed that resveratrol can be genotoxic even at concentrations as low as 5 μM, which can be achieved in laboratory animals by oral administration [10]. The mechanism underlying the genotoxic activity of resveratrol is not well understood. Resveratrol can inhibit DNA polymerases α and δ as well as DNA topoisomerase II [8, 11], so it has been hypothesized that resveratrol induces DNA damage by inhibiting enzymes that participate in DNA replication and/or repair (reviewed in [12]). This hypothesis predicts that cells in the DNA synthesis (S) phase would be the most sensitive to resveratrol.

Activation of DNA-damage signaling leads to activation of the p53 pathway, whose role is to stop the cell cycle and either facilitate DNA repair or permanently inhibit cell growth by inducing cellular senescence or apoptosis. For example, double-strand DNA breaks trigger activation of ATM kinase by autophosphorylation of Ser^1981^; activated ATM then phosphorylates Thr^68^ of CHK2 kinase. Activated CHK2 and ATM both phosphorylate p53 at the N-terminus, thereby stabilizing p53 by preventing its association with MDM2, the ubiquitin ligase and negative regulator of p53 (reviewed in [13]). Activated p53 induces transcription of the gene encoding p21, which is the major effector of cell-cycle inhibition (reviewed in [14]). p53 transcriptionally induces its negative regulators, the MDM2 and WIP1 proteins ([15] and references therein). The latter protein can dephosphorylate and inactivate p53 as well as ATM and CHK2 [16–18]. The p53 pathway can also be activated non-genotoxically by nutlin-3a, an experimental drug designed to bind MDM2 specifically in its p53-binding pocket. Nutlin-3a prevents MDM2 from binding and destabilizing p53, leading to accumulation of high levels of p53 and upregulation of some p53-dependent genes, e.g., p21 and MDM2 [19].

In a previous study, we showed that resveratrol activates the p53 pathway in A549 and U-2 OS cells, as indicated by the accumulation of p53 protein and by the phosphorylation of p53 on Ser^15^ and Ser^37^. However, p21 was efficiently upregulated only in A549 cells, suggesting that the resveratrol-induced activation of the p53 pathway was attenuated in U-2 OS cells when compared with A549 cells. Despite their weak activation of the p53 pathway, U-2 OS cells exposed to resveratrol exhibited senescence-like growth inhibition. Moreover, we noticed strong differences between resveratrol-treated A549 and U-2 OS cells regarding expression pattern of the major cell-cycle regulators, e.g., BRCA1, cyclin B1, and RB [7]. The goal of this study was to explore further the molecular differences between A549 and U-2 OS cells, which could account for the different modes of p53 pathway activation. We conjectured that attenuation of p53 activation in U-2 OS cells might be specific to a subset of stress factors, such as those that trigger extensive post-translational modifications of p53, e.g., DNA damage. Hence, we planned to compare the status of nutlin-3a-induced p53 activation between A549 and U-2 OS cells. Moreover, we wanted to explore how nutlin-3a would modify the response of cells to genotoxic doses of resveratrol. The results of these experiments should help us understand the molecular mechanisms underlying the cytostatic activity of resveratrol, which is still a matter of debate.

## Materials and methods

### Cell culture, treatment, cytometric analysis of DNA content, and clonogenic assays

U-2 OS (human osteosarcoma, ATCC), A549 (human lung adenocarcinoma, ATCC), and GM07492 (normal human fibroblasts, Coriell Cell Repositories, Camden, NJ) cells were grown at 37 °C in an atmosphere containing 5 % CO_2_ in Dulbecco’s modified Eagle’s medium (DMEM, Sigma-Aldrich, St. Louis, MI) supplemented with 10 % (U-2 OS, A549) or 15 % (GM07492) fetal bovine serum (Gibco-Invitrogen, Carlsbad, CA) and penicillin–streptomycin solution (Sigma-Aldrich). GM07492 cells were used between passages 11 and 12.

The following stock solutions of chemicals were used: resveratrol (100 mM in DMSO; Sigma-Aldrich), nutlin-3a (10 mM in DMSO; Cayman Chemical, Ann Arbor, MI). Stock solutions were dissolved in culture medium to obtain the required concentration of the chemicals (50 μM resveratrol or 5 μM nutlin-3a). Control cells were treated with medium containing indicated concentration of DMSO (0.05 or 0.1 %).

Cell-cycle profiles were obtained by FACS analysis (FACSCanto flow cytometer, Becton–Dickinson, Franklin Lakes, NJ) following trypsinization of attached cells, ethanol fixation, RNase treatment, and propidium iodide (PI) staining.

For clonogenic assays, cells were seeded into 6-well plates. Starting the next day, the cells on experimental plates were treated for 96 h with 50 μM resveratrol, 5 μM nutlin-3a, or both substances; the control cells were mock-treated. After treatment, the cells were extensively washed and allowed to recover in fresh medium for 24 h. Subsequently, the cells were trypsinized and counted, and 1,000 cells from each sample were seeded into 6 cm plates containing 5 ml of fresh medium. After incubation for 9 (A549) or 12 (U-2 OS) days, colonies were fixed with 1:1 methanol:acetone and visualized with crystal violet. For each data point, large colonies were counted on two plates from at least three independent experiments.

### Suppression of WIP1 expression by lentivirus-delivered shRNA

The expression of WIP1 protein in U-2 OS cells was suppressed using transduction-ready lentiviral particles purchased from Santa Cruz Biotechnology (Santa Cruz, CA). The manufacturer’s protocol was followed. Control cells were transduced with lentiviral particles (from the same manufacturer) encoding scrambled shRNA particles that do not induce specific degradation of any known mRNA. Positively transduced cells were selected with puromycin. Due to high transduction efficiency, clonal selection was not required. The efficiency of knockdown was monitored by Western blotting. The day before treatment, the cells were trypsinized, seeded into new plates, and incubated in puromycin-free medium. Other resveratrol treatment conditions were as described above.

### Immunofluorescence staining of histone H2AX phosphorylated on serine 139 (γH2AX)

Staining was performed as described previously [7, 20]. Cells on Lab-Tek II slides (NUNC, Roskilde, Denmark) were fixed for 2 min at room temperature with 3.7 % formalin in PBS (Sigma-Aldrich), washed, and then permeabilized in PBS containing 0.5 % Triton X-100 (Sigma-Aldrich) for 10 min. After washing, the cells were incubated in blocking solution (5 % BSA and 0.15 % glycine in PBS) at room temperature for 30 min. After 2 h incubation with anti-phospho-Ser139 histone H2AX antibody (JBW301, Upstate-Millipore, Billerica, MA; diluted 1:500 in blocking solution), the cells were extensively washed and incubated with Texas Red-conjugated anti-mouse IgG antibody (Vector Laboratories, Burlingame, CA), diluted 1:300. The stained cells were embedded in Vectashield with DAPI (Vector Laboratories) and were observed using a Nikon Eclipse E80*i* fluorescence microscope.

### Western blotting

Control and treated cells growing on culture plates were harvested by trypsinization. For preparation of whole-cell lysates, PBS-washed cell pellets were frozen on dry ice and stored at −70 °C. Subsequently, the frozen cell pellets were suspended in IP buffer (50 mM Tris–HCl, pH 8.0; 120 mM NaCl; 0.5 % NP-40) supplemented with protease inhibitors (PMSF, pepstatin A, aprotinin, and leupeptin) and Phosphatase Inhibitor Cocktail 2 (Sigma-Aldrich). After incubation on ice for 20 min, lysates were cleared by centrifugation (14,000 rpm, 4 °C, 20 min). Subsequently, two volumes of cleared lysate was mixed with one volume of solution containing 150 mM Tris (pH 6.8), 6 % SDS, 30 % glycerol, 0.01 % bromophenol blue, and 7.5 % β-mercaptoethanol. Lysates were then denatured (95 °C, 5 min), chilled on ice, and stored at −70 °C.

Nuclear extracts were prepared by a method described previously [7]. After trypsinization and washing with PBS, cell pellets were treated with ice-cold EC buffer (20 mM Tris, pH 7.6; 10 mM KCl; 2 mM MgCl_2_; 1 mM DTT; 0.5 mM EGTA; 0.5 % NP40; 2.5 % glycerol) supplemented with the protease and phosphatase inhibitors mentioned above. The suspension was incubated on ice for 10 min. Subsequently, the samples were centrifuged at 310×*g* at 4 °C for 10 min. The cytoplasmic fractions in the supernatants were discarded, and the pellets enriched in cell nuclei were frozen at −70 °C. After thawing on ice, pellets were lysed on ice for 20 min with RIPA buffer (0.5 % NP40, 0.5 % sodium deoxycholate, 0.1 % SDS in PBS) supplemented with protease and phosphatase inhibitors. After centrifugation and denaturation as described above, the nuclear extracts were stored at −70 °C.

Subsequently, 10–50 μg aliquots of whole-cell lysates or nuclear extracts were separated by 6 or 11 % SDS-PAGE and electrotransferred onto PVDF membranes. The membranes were blocked for 1 h at room temperature in blocking solution (5 % skim milk solution in PBS with 0.1 % Tween-20) and incubated with the indicated primary antibody. The following antibodies were from Cell Signaling Technology: anti-phospho-Ser1981 ATM (D6H9), anti-ATM (D2E2), anti-acetyl-Lys382 p53, anti-phospho-Ser15 p53 (rabbit polyclonal antibody), anti-phospho-Ser20 p53, anti-phospho-Ser37 p53, anti-phospho-Ser392 p53, anti-CHK2 (rabbit polyclonal antibody), anti-phospho-Thr68 CHK2, anti-phospho-Ser807/811 RB, and anti-PLK1 (208G4). Anti-BRCA1 (D-9), anti-CDC2 (17), anti-p53 (DO-1), and anti-p21^WAF1^ (F-5), anti-MDM2 (HDM2-323) antibodies were from Santa Cruz Biotechnology. Anti-retinoblastoma protein (RB) antibody (clone mAB245) was from Chemicon International, and anti-14-3-3σ (Ab14116) and anti-PPM1D (WIP1) antibodies (Ab31270) were from Abcam (Cambridge, UK). HSC70 loading control was detected using the B-6 antibody (Santa Cruz Biotechnology). All incubations with primary antibodies were performed overnight at 4 °C in blocking solution. The secondary antibodies were HRP-conjugated and detected by chemiluminescence.

### Semi-quantitative real-time PCR

Total RNA samples were prepared using the RNeasy mini kit according to the manufacturer’s protocol (Qiagen, Hilden, Germany). cDNAs were synthesized using MuLV reverse transcriptase and random hexamers (Applied Biosystems, Foster City, CA). Measurements of p21, MDM2, PPM1D, and β-actin (internal reference) mRNA levels were performed using Real-Time 2× PCR Master Mix SYBR (A&A Biotechnology, Gdynia, Poland) with oligonucleotide sequences GTG GAC CTG TCA CTG TCT TG and GAT TAG GGC TTC CTC TTG G for p21, GAG ACC CTG GTT AGA CCA AAG C and GCA CGC CAA ACA AAT CTC C for MDM2, CTC AAT GTG CCA GGA CCA AGA G and TAT CTG CTC GGA GCA TAC GCT G for WIP1 (PPM1D), and GCA AGC AGG AGT ATG ACG AG and CAA ATA AAG CCA TGC CAA TC for β-actin mRNA [21]. PCR was performed using a CFX96 Real-Time System (Bio-Rad, Hercules, CA). In each PCR run, the cDNA samples were amplified in triplicate. The relative quantification of the mRNAs for p21, WIP1, and MDM2 was performed using the ∆∆C_T_ method, with β-actin as the reference. Means and standard deviations were calculated from two independent treatments.

### RT-PCR and sequencing of the WIP1 gene (*PPM1D*) coding sequence

RNA and cDNA samples from untreated A549 and U-2 OS cells were prepared as described in the previous section. RT-PCR amplifications in the presence of 6.5 % DMSO were performed using AmpliTaq Gold polymerase (Applied Biosystems); hence, the reactions were started with a 10 min incubation at 95 °C to activate the enzyme. Subsequently, 40 cycles of amplification (94 °C, 30 s; 58 °C, 15 s; 72 °C, 75 s) were performed. The *PPM1D* cDNA sequence was amplified as two overlapping fragments encompassing the coding region. The primer sequences are given in the 5′→3′ direction. The 5′ fragment (1,012 bp) was amplified with the following primers: WIP-P1, GGC GTC GTC GAA GAT AAA CA; WIP-RT1, GTC AAG AGT GTG GAC ACT TG. The 3′ fragment (1,116 bp) was amplified with the following primers: WIP-RT2, TTG TGG TGT CACCTG AAC CAG; WIP-P12, CAA GCA AGT ACA AGG CCA GGA. Subsequently, the PCR products were prepared for sequencing by exonuclease I and shrimp alkaline phosphatase digestion of unincorporated primers and deoxynucleotides. Prepared PCR products were sequenced using DigDye Terminator v3.1 Cycle Sequencing Kit (Applied Biosystems), and the sequences were acquired using a Genetic Analyzer 3500 (Applied Biosystems). The 5′ fragment was sequenced using the following primers: WIP-SQ1, AAC AAT AGT TGG CCG GCG AG; WIP-RT3, AGA AGG GTT TCA CCT CGT CC. The 3′ fragment was sequenced using the following primers: WIP-RT4, GAG GGT ATG ACT ACA CCT TG; WIP-SQ12, GTT CAA CAT CGG CAC CAA AT. Genomic DNA from A549 and U-2 OS cells was isolated by the chloroform extraction and ethanol precipitation method using the Genomic DNA Purification kit (Fermentas, Vilnius, Lithuania). Exon 6 of *PPM1D* was amplified with the following primers: WIP-P11, CAA GCA AGT ACA AGG CCA GGA; WIP-P12 (see above). Exon 6 was amplified from genomic DNA as a template using AmpliTaq Gold polymerase in the presence of 5.2 % DMSO. Thirty-five cycles of amplification (94 °C, 30 s; 60 °C, 30 s; 72 °C, 45 s) were performed. The fragment was sequenced as described above with the following primers: WIP-SQ11, TCA CAT GCA TAG ATT TGT TGA GTT C; WIP-SQ12 (see above).

## Results

### U-2 OS cells exposed to resveratrol exhibit attenuated upregulation of mRNA and proteins encoded by p53 target genes

**Fig. 1:**
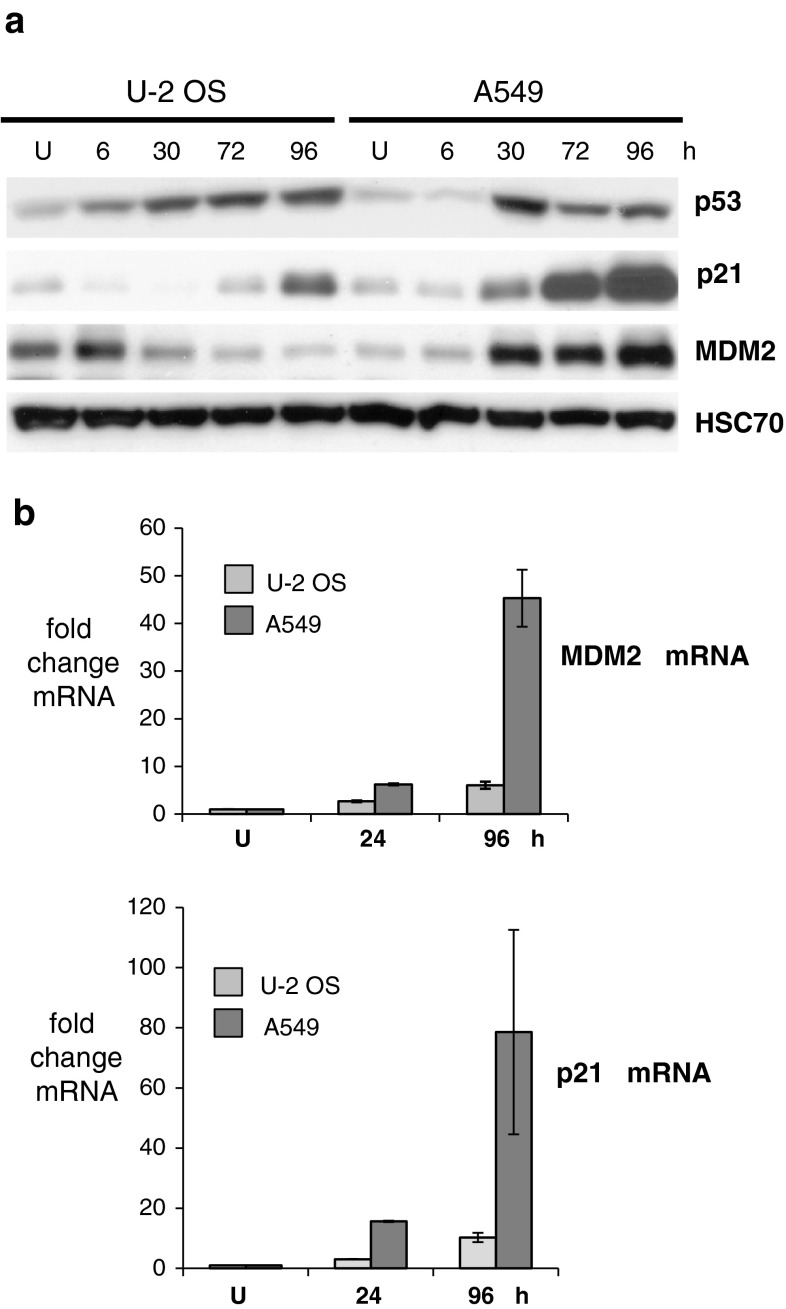
**a** Expression of p53 and proteins encoded by p53-regulated genes (p21 and MDM2). Whole-cell lysates of untreated cells (U) and of cells treated for the indicated number of hours with 50 μM resveratrol were analyzed. HSC70 is a loading control. **b** Changes in the levels of mRNAs encoding MDM2 or p21, measured by semi-quantitative real-time PCR in RNA samples isolated from untreated (U) cells and from cells treated with resveratrol for 24 or 96 h. The results represent the means and standard deviations from two independent experiments

**Fig. 2:**
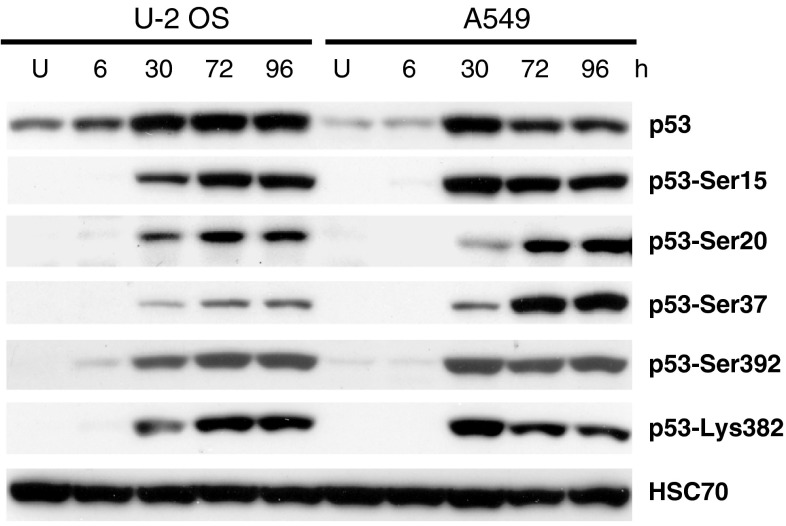
Expression of p53 and its post-translationally modified forms in whole-cell lysates from untreated cells (U) or from cells treated with resveratrol for the indicated number of hours in a time-course experiment. Phosphorylation of serines 15, 20, 37, and 392 and acetylation of lysine 382 were examined

Despite accumulation of p53 protein induced by resveratrol, U-2 OS cells exhibited attenuated upregulation of p53 target genes (e.g., those encoding p21 and MDM2) when compared with A549 cells. This attenuation was visible at both the protein (Fig. 1a) and mRNA levels (Fig 1b). Potentially, insufficient post-translational modification of p53 could be the source of the apparently weaker p53 activity in U-2 OS cells. Previously, we found that in both cell lines, p53 was phosphorylated at Ser^15^ and Ser^37^, although the level of p53 with phosphorylated Ser^37^ was slightly higher in A549 cells [7]. This observation was confirmed in this study both in nuclear extracts (not shown) and in whole-cell lysates prepared in a separate experiment (Fig. 2). Moreover, in this study we observed that the other crucial modifications of p53 (phosphorylation of Ser^20^ and Ser^392^ as well as acetylation of Lys^382^) were similar in both cell lines (Fig. 2). Thus, the source of relatively weak p53 pathway activation in resveratrol-treated U-2 OS cells has not been determined, although it is associated with the lower phosphorylation level of Ser^37^. We hypothesize that the reduced ability of p53 to activate its target genes in U-2 OS cells could result from deficiencies in post-translational modifications of residues that were not studied by us, and/or from altered activities of proteins functionally associated with p53.

### The resveratrol-induced cell-cycle inhibition of A549 cells is associated with strong downregulation of proteins involved in regulation of the G2/M checkpoint

**Fig. 3:**
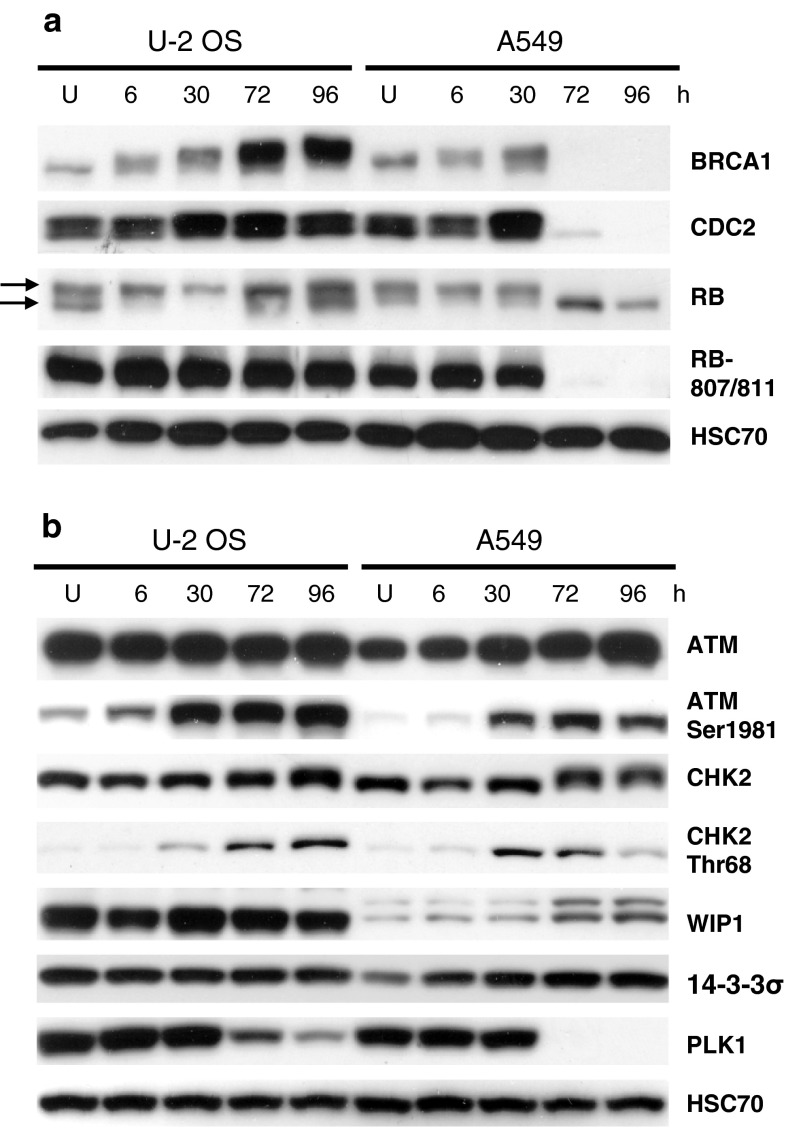
**a** Expression of crucial cell-cycle regulators in nuclear extracts of untreated cells (U) or cells treated with resveratrol for the indicated number of hours. RB-807/811 shows the phosphorylation of Ser^807^ and Ser^811^ of RB. The *upper* and *lower* *arrows* show, respectively, the hyper- and hypophosphorylated forms of RB. **b** Expression and phosphorylation status of kinases activated by DNA damage (ATM and CHK2), expression of the phosphatase acting on them (WIP1), and expression of PLK1 kinase in whole-cell lysates of cells treated as in **a.** 14-3-3σ is an important regulator of G2/M progression

The mechanistic basis of cell-cycle inhibition in U-2 OS cells exposed to resveratrol is not known [7]. The low activity of p21 in resveratrol-treated U-2 OS cells manifests as the persistent phosphorylation of RB protein (Fig. 3). Accumulated p21 inhibits RB phosphorylation (reviewed in [22]). Previously, we found that the different degrees of p53 pathway activation between A549 and U2OS cells were associated with differences in the expression levels of crucial cell-cycle regulators [7]. Strong upregulation of p21 in A549 was associated with repression of BRCA1, cyclin B1, and the phosphorylated form of RB. In this study, we expanded this analysis to other regulators of the G2/M checkpoint (Fig. 3). Using an antibody that specifically recognizes the phosphorylation of RB at Ser^807^ and Ser^811^, we confirmed the inhibition of RB phosphorylation in A549 cells. Moreover, in these cells we detected strong repression of cyclin-dependent kinase CDC2 (CDK1), which, in complex with cyclin B1, is the major kinase that induces the G2/M transition. Interestingly, A549 cells also exhibited strong repression of another kinase, PLK1, which is crucial for entering mitosis (reviewed in [23]). Thus, unlike U-2 OS cells, resveratrol-treated A549 cells exhibited coordinated repression of major regulators of cell-cycle progression (cyclin B1, CDC2, BRCA1, phosphorylated RB, and PLK1). The cell-cycle inhibition of A549 cells treated with resveratrol can be explained in molecular terms: they cannot progress to mitosis due to a shortage of activated kinases (CDC2/cyclin B1, PLK1) that are crucial for starting cell division (reviewed in [23]). The mechanism underlying cell-cycle inhibition of U-2 OS cells exposed to resveratrol is unknown; furthermore, these cells also do not upregulate 14-3-3σ, the product of another p53 target gene involved in G2 arrest [24] (Fig. 3b).

### Cell-cycle inhibition of resveratrol-treated U-2 OS cells is associated with persistent signaling through the ATM pathway

It is plausible that U-2 OS cells have an unidentified defect in p53 activation that leads to relatively weak upregulation of target genes during resveratrol exposure. Hypothetically, such a defect might involve a mechanism upstream of the p53 signaling pathway. In response to DNA damage, p53 is phosphorylated by the ATM and CHK2 kinases (reviewed in [25]). CHK2 is activated by ATM-mediated phosphorylation [26]. To examine the activation status of these p53 regulators, we monitored the phosphorylation of ATM Ser^1981^ and CHK2 Thr^68^. The ATM pathway was activated in both cell lines. In A549 cells, CHK2 phosphorylation peaked at the 30 h time point and diminished after 72 and 96 h (Fig. 3b). By contrast, in U-2 OS cells, CHK2 phosphorylation peaked after 96 h. Thus, the signaling through the ATM-CHK2 pathway differed between the two cell lines: A549 cells exhibited activation and subsequent repression of the signaling, whereas in U-2 OS cells the signaling persisted for 96 h. Similar patterns of H2AX phosphorylation have been reported previously [7].

### U-2 OS cells overexpress *PPM1D* gene, which encodes a negative regulator of the p53 pathway, WIP1

We hypothesized that repression of CHK2 phosphorylation in A549 cells at later time points of resveratrol treatment was the result of strong activation of the p53 pathway. This conjecture is based on data reported by others, who found that WIP1 protein, the product of a p53-activated gene (*PPM1D*), is able to dephosphorylate CHK2 [18]. Hence, we analyzed the dynamics of WIP1 expression in whole-cell lysates of U-2 OS and A549 cells treated with resveratrol. As predicted, in A549 cells, WIP1 was upregulated at the 72 and 96 h time points. Surprisingly, however, in U-2 OS cells, WIP1 protein was overexpressed, and its expression did not exhibit major changes in response to resveratrol treatment (Fig. 3b).

**Fig. 4:**
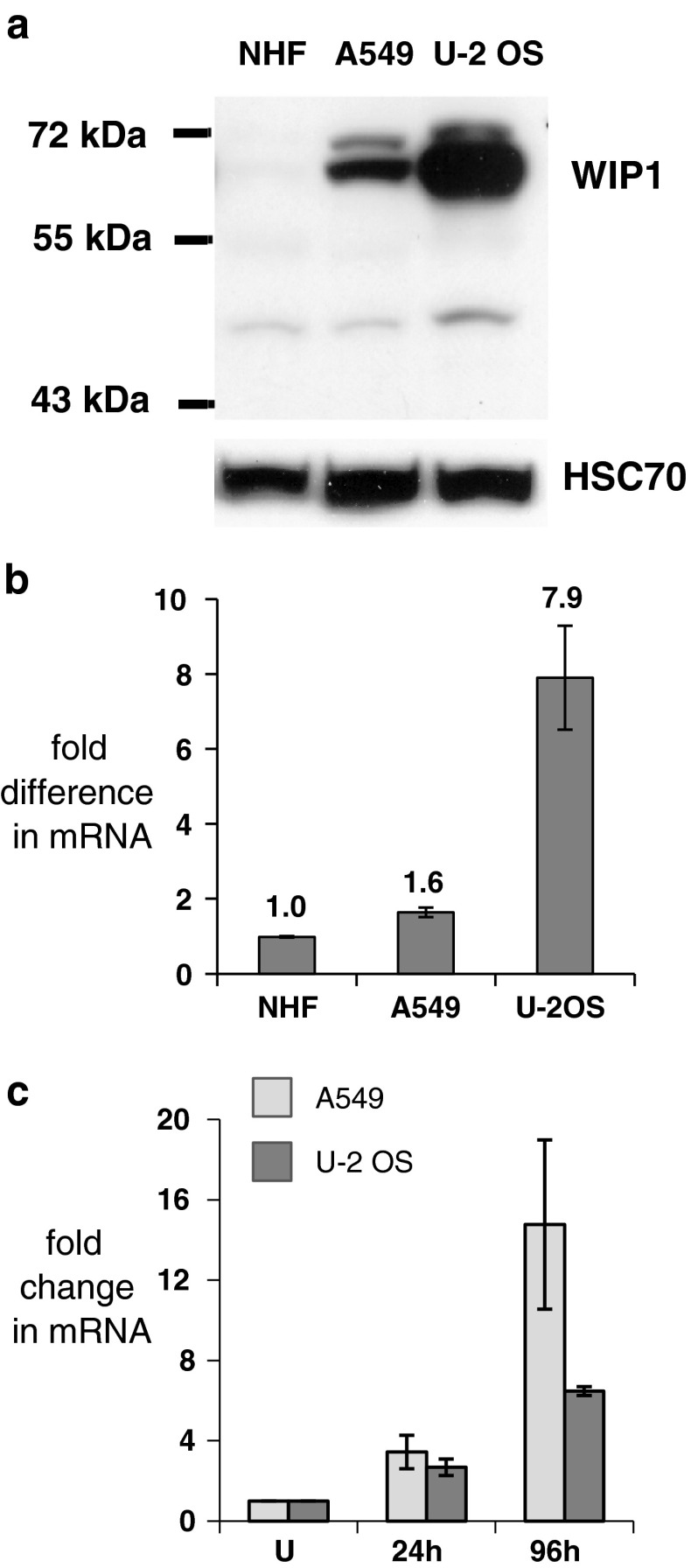
**a** The expression of WIP1 protein in whole-cell lysates isolated from untreated normal human fibroblasts (NHF; GM07492) and A549 or U-2 OS cancer cell lines. The position of molecular-weight markers is shown on the *left*. **b** Expression of mRNA encoding WIP1, measured by semi-quantitative real-time PCR in RNA samples from the cells shown in (**a**). The results represent the means and standard deviations from two independently isolated RNA samples. **c** Changes in the levels of mRNA encoding WIP1, measured by semi-quantitative real-time PCR in RNA samples isolated from untreated cells (U) or from cells treated for 24 or 96 h with resveratrol. The level in the untreated population of either cell line was defined as 1. The results represent the means and standard deviations from two independent experiments

**Fig. 5:**
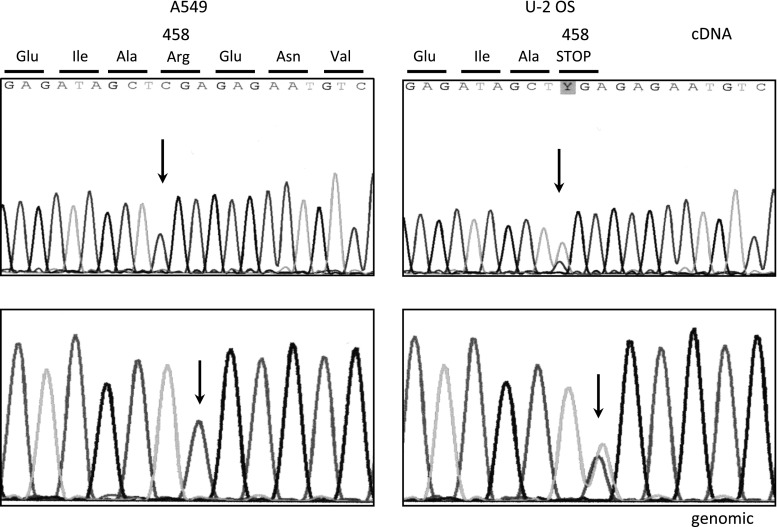
Electropherogram showing a mutation in the gene encoding WIP1 (*PPM1D*) detected in the cDNA of U-2 OS cells (*right*, *upper panel*). The *upper-left panel* shows the relevant fragment of an electropherogram from wild-type cDNA sequence detected in A549 cells. The position of the mutant nucleotide is indicated by *arrows*. Note that in the electropherogram of U-2 OS cells, both wild-type and mutant sequences are visible, indicating that both forms of mRNA are expressed. The mutation 1604:C→T (coordinates according to GenBank sequence, under Accession NM_003620) generates a stop codon in place of amino-acid residue 458. The *lower panels* present the relevant electropherograms of the genomic sequence, zoomed in on the mutant residue

Analysis of WIP1 expression in whole-cell lysates from untreated A549 and U-2 OS cells, as well as those from normal human fibroblasts (GM07492), revealed the lowest expression in fibroblasts, intermediate expression in A549, and the highest expression in U-2 OS cells (Fig. 4a). Consistent with this, the expression of *PPM1D* mRNA measured by semi-quantitative RT-PCR was about eight times higher in U-2 OS cells than in fibroblasts (Fig. 4b). Moreover, *PPM1D* mRNA expression increased in both U-2 OS and A549 cell lines treated with resveratrol, but the magnitude of the increase was greater in A549 cells (Fig. 4c), further supporting the hypothesis that activation of p53 pathway is attenuated in U-2 OS. High expression of the *PPM1D* gene in U-2 OS cells is consistent with its role in carcinogenesis. *PPM1D* is considered to be a proto-oncogene due to its role in negative regulation of p53 and its overexpression in breast, gastric, and pancreatic cancers, as well as in other tumors (reviewed [15]). To exclude the possibility that U-2 OS cells express a mutant form of the gene, we sequenced the *PPM1D* cDNA amplified by RT-PCR. Unexpectedly, we found a nucleotide substitution (Fig. 5). This C→T transition at position 1604 of the reference sequence NM_003620 generates a nonsense mutation in codon 458 (458:CGA→TGA, Arg → STOP). The location of C→T substitution at a CpG dinucleotide is consistent with deamination of 5-methylcytosine as the mutagenic mechanism [27]. This mutation was also visible in heterozygous configuration in the genomic DNA of U-2 OS cells (Fig. 5) indicating that the wild-type and mutant alleles of *PPM1D* are present in equal copy numbers. We have not detected any other mutations in the coding regions of cDNAs from U-2 OS cells. Because these cells contained a major alteration in the gene encoding WIP1 protein, we hypothesized that the attenuated activation of the p53 pathway in U-2 OS cells is caused by overexpression of WIP1.

### Knockdown of WIP1 protein in U-2 OS cells helps to activate p53

**Fig. 6:**
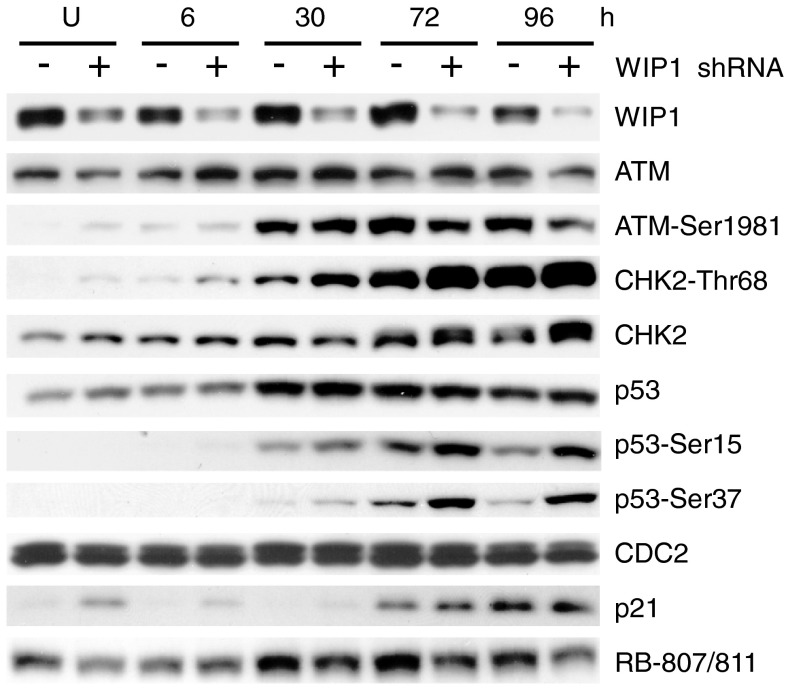
Expression of DNA-damage signaling proteins (ATM, CHK2), their phosphorylated forms, p53, and post-translationally modified p53, as well as the major cell-cycle regulators (p21, CDC2, RB phosphorylated on Ser807/811), examined in whole-cell lysates from untreated U-2 OS cells (U) or from cells treated with resveratrol for the indicated number of hours in a time-course experiment. The pairs represent control cells (−) and WIP1-knockdown cells (+); knockdowns were performed using lentivirus-delivered shRNA molecules. The efficiency of knockdown is shown by immunodetection of WIP1 protein

To determine whether the increased expression of WIP1 in U-2 OS cells is the cause of the relatively weak activation of the p53 pathway, we performed a time-course experiment on cells in which WIP1 had been knocked down. The knockdown was performed using lentiviral particles containing three specific constructs encoding 19–25 nt shRNAs targeting the *PPM1D* mRNA. Control cells were transduced with control lentivirus. The successful knockdown of WIP1 in U-2 OS did not result in the same molecular response to resveratrol observed in A549 cells, i.e., neither downregulation of RB phosphorylation nor repression of CDC2 was observed (Fig. 6). However, at early time points of resveratrol treatment or in untreated cells, WIP1 knockdown resulted in slightly increased expression of p21 and phosphorylated CHK2 (on Thr^68^). This effect was reproducibly observed in independent experiments. Moreover, knockdown of WIP1 resulted in stronger resveratrol-induced phosphorylation of p53 on Ser^37^. To the best of our knowledge, this is the first study showing that WIP1 expression modulates the phosphorylation of p53 on this residue. We also observed an influence of WIP1 repression on p53 Ser^15^ phosphorylation. Thus, downregulation of WIP1 in U-2 OS cells helps to activate p53, but does not result in major changes in expression of the cell-cycle regulators we examined (RB, CDC2).

### Nutlin-3a upregulates the p53 pathway in both U-2 OS and A549 cell lines and induces the accumulation of G1 phase cells in resveratrol-treated populations

**Fig. 7:**
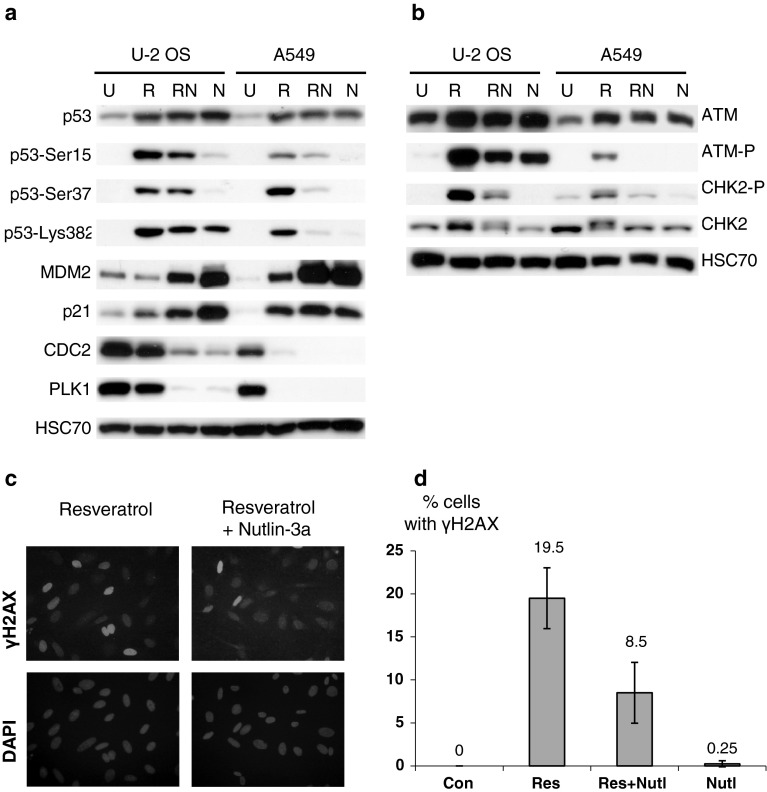
**a** Expression of the indicated proteins in whole-cell lysates of cells treated for 96 h with 50 μM resveratrol (R), 5 μM nutlin-3a (N), or co-treated with both substances (RN). **b** The expression of DNA-damage signaling proteins (ATM and CHK2) and their phosphorylated forms (ATM-Ser1981, CHK2-Thr68) in whole-cell lysates of cells treated as in **a**. **c** Immunocytochemical staining of histone H2AX phosphorylated on Ser^139^ (γH2AX) in U-2 OS cells treated for 96 h with resveratrol or with resveratrol and nutlin (upper photographs). The locations of cell nuclei were visualized by DAPI staining. **d** The frequency of U-2 OS cells with upregulated γH2AX; cells were treated as in **a**. Staining was performed after 96 h treatment. Means and standard deviations are shown. At least 400 cells in two independent experiments were examined

**Fig. 8:**
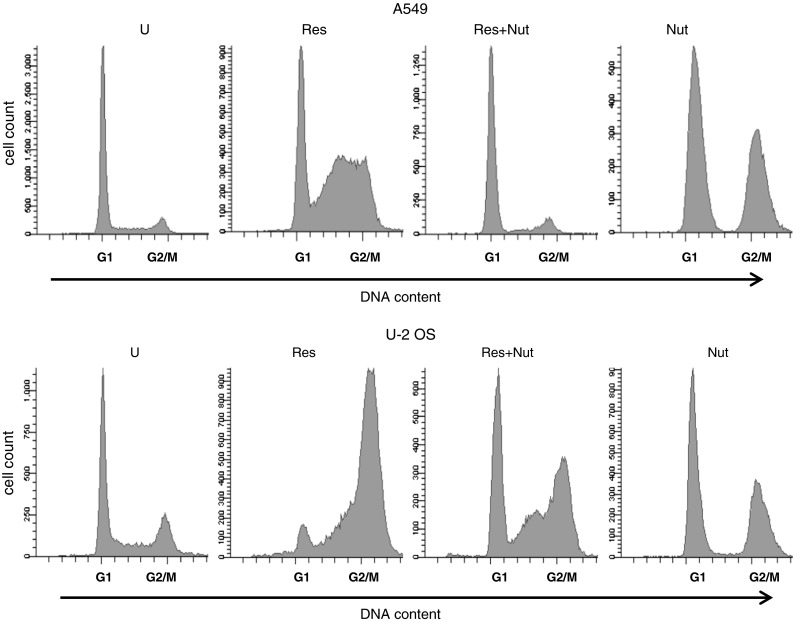
The cell-cycle distribution of untreated cells (U) or cells exposed to resveratrol (Res), nutlin-3a (Nut), or the combination of both substances (Res+Nut) for 96 h. The *horizontal axis* shows DNA content. The values for G1- and G2/M-phases are marked. The *vertical axis* shows the cell count

To examine the mechanism of p53 activation in U-2 OS cells more closely, we decided to determine whether the p53 pathway could be activated by a non-genotoxic agent, nutlin-3a, which apparently activates p53 in a way that does not require extensive phosphorylation of p53 on key serine residues [28]. In both cell lines, treatment with nutlin-3a resulted in upregulation of p53 and accumulation of the major p53 targets, p21 and MDM2 (Fig. 7a). As expected, p53 activation induced by nutlin-3a was not associated with strong phosphorylation of p53 at Ser^15^ and Ser^37^. For unknown reasons, U-2 OS exhibited increased acetylation of Lys^382^. Thus, in contrast to resveratrol, nutlin-3a has the ability to activate the p53 pathway in U-2 OS strongly. Because nutlin-3a and resveratrol activated the p53 pathway by different mechanisms, we next asked how nutlin-3a modulates the resveratrol-induced activation of p53 and DNA-damage signaling pathways. To the best of our knowledge, this has not been previously addressed in regard to resveratrol treatment, although earlier studies have described how nutlin-3a modulates the biological and molecular consequences of treating cells with various other genotoxic agents (see Discussion). In A549 cells, nutlin-3a attenuated p53 modifications induced by resveratrol (Fig. 7a). This effect was not visible in U-2 OS cells (two independent experiments showed this phenomenon). Based on these results, we hypothesized that in A549 cells, nutlin-3a attenuates signaling through the ATM pathway, resulting in lower levels of p53 modifications at Ser^15^ and Ser^37^. To test this hypothesis, we looked for signs of ATM pathway activation (phosphorylation of ATM at Ser^1981^ and of CHK2 at Thr^68^) in cells treated as in Fig. 7a. The results were consistent with our hypothesis (Fig. 7b). Nutlin-3a diminished the ATM phosphorylation in A549 cells treated with resveratrol. In U-2 OS cells, the major sign of nutlin-induced ATM pathway downregulation was lower phosphorylation of CHK2. To support further the notion that the resveratrol-induced DNA-damage signaling in U-2 OS cells is reduced by nutlin-3a, we determined the percentage of cells containing high levels of γH2AX. After 96 h, the frequency of γH2AX-overexpressing cells was significantly reduced in U-2 OS cells co-treated with both substances, relative to cells exposed only to resveratrol (Fig 7c, d). Moreover, we observed higher MDM2 expression in cells co-treated with resveratrol and nutlin-3a than in cells treated only with resveratrol (Fig. 7a). These findings are consistent with observations that high activity of DNA damage-activated kinases destabilizes MDM2 [29]. Based on these results, we conclude that nutlin-3a has the ability to reduce the activation of DNA-damage signaling. To explain this, we hypothesized that nutlin somehow prevents the formation of DNA damage in cells exposed to resveratrol. Some observations indicate that resveratrol induces DNA damage mostly in cells that are replicating their DNA (reviewed in [12]). If nutlin-3a prevents the cells from entering the S phase of the cell cycle, it might diminish the induction of DNA damage by resveratrol. The results of a cytometric analysis were consistent with this hypothesis (Fig. 8). As we previously reported [7], resveratrol-treated cells accumulated in S or late S/G2 phase of the cell cycle. Nutlin-3a alone blocked both cell lines either in G1 or in G2 (Fig. 8), consistent with data published by others [30]. S phase cells were virtually absent in populations treated with nutlin-3a for 96 h. In co-treated cell populations, the frequency of cells in G1 phase significantly increased in both cell lines. Thus, diminished ATM pathway activation resulting from nutlin-3a co-treatment is associated with inhibition of the cell cycle before the DNA replication phase.

Nutlin-3a treatment also helped us to identify the likely source of differences in the expression patterns of PLK1 and CDC2 between U-2 OS and A549 cells exposed to resveratrol for extended periods of time (Fig. 3). Based on data published by others showing that the genes encoding CDC2 and PLK1 are repressed by p53 [31, 32], we conjectured that repression of CDC2 and PLK1 in A549 cells, and their lack of repression in U-2 OS cells, were consequences of differences in the activation status of the p53 pathway. Indeed, this apparently was the case. Nutlin-3a alone strongly repressed PLK1 and CDC2 in A549 cells (Fig. 7a). Moreover, nutlin-3a was able to repress the two proteins in U-2 OS cells, even though these cells did not repress these proteins in response to resveratrol treatment. We conclude that when U-2 OS cells are treated with resveratrol, p53 can neither strongly upregulate p21 and MDM2 nor strongly repress PLK1 and CDC2; however, when p53 is activated by nutlin-3a, it efficiently activates and represses the p53-regulated genes.

### Nutlin-3a helps to preserve the replication potential of resveratrol-treated cells

**Fig. 9:**
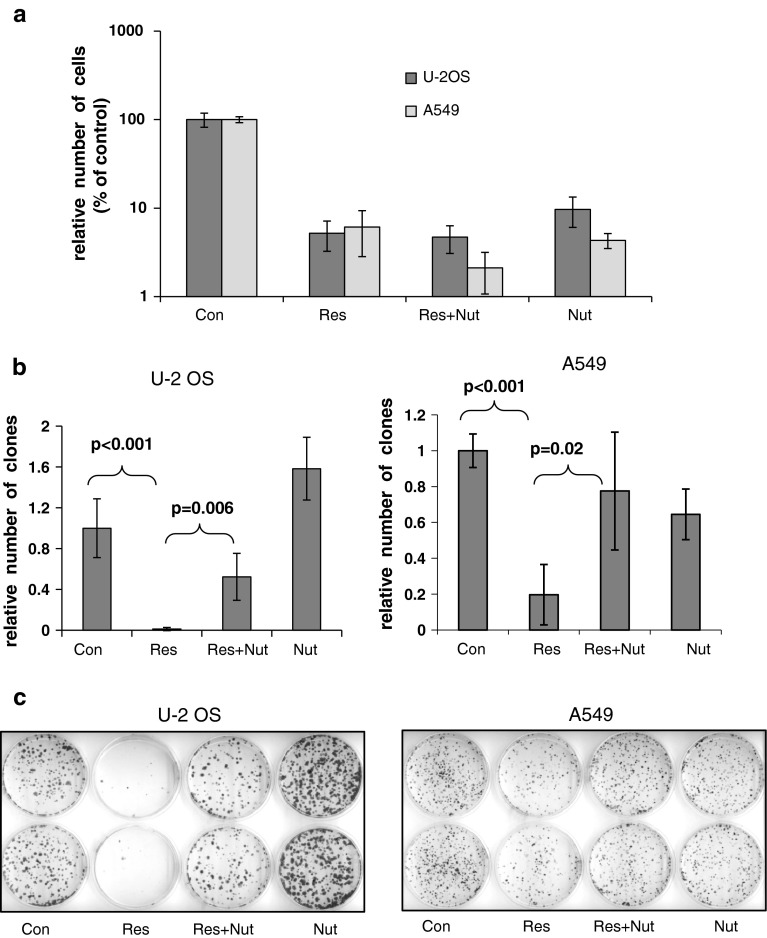
**a** Growth-inhibitory effect of resveratrol and nutlin-3a in U-2 OS and A549 cells in a short-term assay. Equal numbers of cells were seeded onto culture dishes at the start of the experiment. The next day, control cells were mock-treated while experimental cells were treated with resveratrol, nutlin, or a combination of both substances. After 96 h, the attached cells were washed, trypsinized, and counted. The graph was drawn from means and standard deviations from two independent experiments performed in triplicate. The number of cells on control dishes after 96 h culture is considered as 100 %. Note that a logarithmic scale is used. **b** The effects of resveratrol, nutlin, and the combination of both substances on the clonogenic potential of U-2 OS and A549 cells. The *vertical axis* shows the relative number of clones, which was defined as 1 for the untreated cells. The *graphs* show the means and standard deviations from three (U-2 OS) or four (A549) independent experiments. The *t* test for two samples was used to determine statistical significance. **c** Example of plates from the clonogenic experiment. Colonies on 6 cm plates were stained with crystal violet

Next, we asked whether nutlin-3a could modify the fate of resveratrol-treated cells. Both nutlin-3a and resveratrol, acting alone or in combination, were able to inhibit the growth of both cell populations after 96-h treatments (Fig. 9a). Previously, we showed that resveratrol significantly decreased the clonogenic potential of both cell lines. This finding, together with the morphological and biochemical characteristics of the treated cells, indicated the induction of senescence-like growth inhibition [7]. In this study, we found that even though nutlin strongly inhibited growth of the cell population (Fig. 9a), it did not induce a very strong change in the clonogenic potential of cells (Fig. 9b, c). Consistent with previous results, we found that resveratrol significantly reduced the clonogenicity of both cell lines, although we noticed that U-2 OS cells were slightly more sensitive in this assay. Unexpectedly, however, nutlin-3a helped to preserve the replication potential of resveratrol-treated cells of both lines (Fig. 9b, c). Thus, even though both resveratrol and nutlin-3a could activate p53, only resveratrol strongly reduced clonogenic potential. Moreover, nutlin-3a had a dominant effect over resveratrol with respect to influence on clonogenic growth potential. Thus, increased activation of the p53 pathway induced by nutlin-3a did not reduce growth, but instead helped to preserve growth potential, in cells that were transiently treated with genotoxic dose of resveratrol.

## Discussion

The results of this study demonstrate that the p53 pathway, activated by the non-genotoxic agent nutlin-3a, can counteract the loss of replicative potential induced by a genotoxic dose of resveratrol. The protective mechanism of nutlin-3a may be complicated, but our observations are consistent with the hypothesis that nutlin-3a arrests the cell cycle at a phase when the cells are less sensitive to the genotoxic activity of resveratrol. Nutlin-3a markedly increases the frequency of resveratrol-treated cells arrested with G1-phase DNA content (Fig. 8). Moreover, nutlin-3a reduces the activation level of the DNA-damage signaling pathway (ATM, CHK2, and histone H2AX). We speculate that resveratrol induces replication stress, e.g., by inhibiting the activity of DNA polymerases [11]. This in turn elevates DNA-damage signaling, p53 activity, and stress-induced senescence, leading to low clonogenic potential. When treated with nutlin-3a, cells preferentially arrest before or after the DNA replication phase, thereby decreasing the chance of DNA damage formation by resveratrol. Consequently, activation of DNA-damage signaling is low, and cells have normal replicative potential when nutlin-3a and resveratrol are removed from the culture medium.

Our hypothesis is consistent with the observations of Kranz and Dobbelstein [30], who revealed that nutlin-3a protects cells against the S phase-specific chemotherapeutic agent gemcitabine. The same authors found that nutlin-3a pretreatment increased long-term survival and decreased apoptosis in U-2 OS cells irradiated with UV. A similar protective effect of nutlin-3a was observed in keratinocytes [33]. Moreover, Carvajal et al. [34] found that nutlin-3a pretreatment conferred protection of normal fibroblasts or cancer cells with wild-type p53 against the cytotoxicity of paclitaxel. The results of our study and the aforementioned papers may be relevant to understanding the mechanisms underlying the radio- and chemo-resistance of some cancer cells. If p53 is activated by a non-genotoxic mechanism, it may help cancer cells to survive treatment with S phase- or M phase-specific agents and restart cell divisions when the exposure ends. Thus, p53-negative tumors may be more sensitive to some drug combinations, as long as cancer cells are able to undergo p53-independent death.

Our findings and the results of others [30, 33, 34] apparently contrast with data showing that nutlin-3a sensitizes cancer cells to radiation [35, 36] or potentiates the cytotoxic activity of anti-cancer drugs in solid tumors [37]. These discrepancies may result in part from the different kinetics of nutlin-3a treatment, and/or measurements of different endpoints, in each study. For example, Cao et al. [35] showed by clonogenic assay that nutlin-3a sensitized a lung-cancer cell line to radiation. However, the nutlin-3a treatment was started just before irradiation and was continued 48 h afterward, so it is possible that nutlin-3a did not have time to prevent cells from entering the most sensitive phase of the cell cycle. A similar radiosensitizing effect of nutlin-3a in a clonogenic assay of laryngeal carcinoma cells was detected by Arya et al. [36]; again, however, the nutlin-3a treatment was performed short time (30 min) before irradiation.

The protective role of nutlin-activated p53 shows some similarity to the results of in vivo experiments, in which p53 activity in mice was elevated either by the expression of an extra copy of the p53 gene [38] or through genetic downregulation of the MDM2 protein [39]. These genetically engineered mice exhibited decreased cancer incidence without signs of accelerated aging. Thus, p53 moderately overexpressed in a non-genotoxic way can extend lifespan. The suppression of cellular senescence by p53 under some experimental conditions has also been observed by others [40]. A protective role of p53 has also been suggested by the aforementioned authors, who found that nutlin-3a pretreatment protected against UV-induced toxicity [33]. Thus, accumulating evidence suggests that p53, at least under specific circumstances, acts as a survival factor.

We observed WIP1 overexpression in U-2 OS cells. This was not surprising, because WIP1 gene amplification/overexpression has been detected in adenocarcinomas of the breast, ovary, and pancreas, in gastric carcinomas, and in other cancers, where it was usually associated with poor prognosis (review in [15]). WIP1 knockout mice are viable and cancer-resistant, but they display a range of abnormalities in male individuals including runting, reproductive organ atrophy, reduced fertility, and reduced longevity. Knockout mice of both sexes suffer from immunological abnormalities (reviewed in [15]).

In our study, knockdown of WIP1 increased phosphorylation of CHK2 on Thr^68^, consistent with the finding of Fujimoto et al. [41], who showed that WIP1 is able to dephosphorylate this residue. Moreover, we found that the knockdown resulted in higher phosphorylation of p53 on Ser^37^ and higher expression of p21. Elevated levels of p21 in cells from WIP1 knockout mice have also been observed by others [42]. Thus, overexpressed WIP1 in U-2 OS cells contributes to attenuated activation of the p53 pathway, at least in part by reducing (directly or indirectly) the phosphorylation of p53 molecules on Ser^37^. To the best of our knowledge, this is the first study showing that WIP1 contributes to decreased phosphorylation of this residue.

Unexpectedly, we detected a mutant version of *PPM1D* gene in U-2 OS cells. To the best of our knowledge, this is the first reported point mutation of this gene. In vitro mutagenesis experiments performed by others showed that the WIP1 protein truncated at the C-terminus (composed of amino acids 1–375) is still able to inhibit phosphorylation of Thr^68^ of CHK2 protein [43]. Humans express an alternatively spliced *PPM1D* mRNA in their lymphoid tissues and in testes; this alternative form encodes amino acids 1–420 plus an additional 10 amino acids that are not present in the major isoform of WIP1. The alternative WIP1 also has enzymatic activity [44]. These results suggest that if a C-terminally truncated mutant protein is expressed, it can still carry out some of the wild-type functions. However, we do not have evidence that the protein truncated by the mutation we found is present in cells at a detectable level; indeed, we have some reason to believe that it is not. Because the mutation shortens the reading frame by 25 %, the expected molecular weight of the mutant protein is 50 kDa. However, using the anti-WIP1 antibody, we have not detected any protein of molecular weight lower than wild-type WIP1 (66.7 kDa) or higher than 43 kDa that was present specifically in U-2 OS cells (Fig. 4). The immunogen for this antibody was derived from within residues 200–300 of human WIP1, so it should be able to detect the truncated version of the protein. Hence, we speculate that the mutant gene encodes an unstable protein, whereas for reasons yet to be determined, the wild-type allele of the gene in U-2 OS cells produces an amount of WIP1 that is larger than that in A549 cells.

It is surprising to find a mutation that generates a stop codon in an overexpressed oncogene. Is it a coincidence that U-2 OS cells harbor a nonsense mutation in *PPM1D* and also apparently overexpress the encoded protein from the wild-type allele, or are these two phenomena somehow related? Are *PPM1D* point mutations common in human cancers? Further research is warranted to answer these important questions.

In conclusion, we report that resveratrol at 50 μM concentration activates DNA-damage signaling and the p53 pathway, as well as reduces the replicative potential of cancer cell lines. The upregulation of p53 by the nutlin-3a in resveratrol-exposed cells reduces the activation of DNA-damage signaling, increases the frequency of cells in the G1 phase of the cell cycle, and prevents the loss of replicative potential. These findings indicate that the genotoxic and cytostatic activities of resveratrol depend on cell-cycle progression. The activation of the p53 pathway in resveratrol-exposed U-2 OS cells is attenuated relative to that observed in A549 cells. U-2 OS cells exhibit increased expression of the p53 antagonist PPM1D, but shRNA-mediated knockdown of this protein only slightly increases the activation of p53. Unexpectedly, in U-2 OS cells, one allele of *PPM1D* gene contains a nonsense mutation. The molecular consequences of this mutation will be a subject of future investigations.
